# Efficient cross-protection against serotype 4/8a fowl adenoviruses (FAdVs): recombinant FAdV-4 with FAdV-8a Fiber

**DOI:** 10.1128/spectrum.02462-23

**Published:** 2023-11-15

**Authors:** Yixuan Lu, Yaqin Yuan, Huiru Jiang, Zhenqi Xu, Yiwen Guo, Xudong Cao, Tuofan Li, Zhimin Wan, Hongxia Shao, Aijian Qin, Quan Xie, Jianqiang Ye

**Affiliations:** 1 Key Laboratory of Jiangsu Preventive Veterinary Medicine, Key Laboratory for Avian Preventive Medicine, Ministry of Education, College of Veterinary Medicine, Yangzhou University, Yangzhou, Jiangsu, China; 2 Jiangsu Co-Innovation Center for Prevention and Control of Important Animal Infectious Diseases and Zoonoses, Yangzhou, Jiangsu, China; 3 Joint International Research Laboratory of Agriculture and Agri-Product Safety, The Ministry of Education of China, Yangzhou University, Yangzhou, Jiangsu, China; 4 Institutes of Agricultural Science and Technology Development, Yangzhou University, Yangzhou, Jiangsu, China; Institute of Microbiology Chinese Academy of Sciences, Beijing, China

**Keywords:** FAdV-4, FAdV-8a, Fiber, recombinant virus, attenuation, protection

## Abstract

Recently, the infection of fowl adenoviruses (FAdVs) in chicken flocks has caused huge economic losses to the poultry industry in China. Although immunization with Fiber protein can provide autogenous protection of FAdVs, no commercial multivalent vaccines are available for the prevention and control of FAdVs. In this study, a novel recombinant virus FAdV4-F/8a-rF2 expressing the Fiber of FAdV-8a was generated through CRISPR-Cas9 and Cre-LoxP system by using the template virus FA4-EGFP. FAdV4-F/8a-rF2 not only exhibited a similar replication capacity to the wild-type FAdV-4 in Leghorn male hepatoma (LMH) cells but was also highly attenuated to specific pathogen free (SPF) chickens. Moreover, the inoculation of FAdV4-F/8a-rF2 could induce high neutralizing antibodies and provide full protection against both FAdV-4 and FAdV-8a. All these data demonstrate that the recombinant virus FAdV4-F/8a-rF2 developed here can be used as an attenuated bivalent vaccine candidate for the prevention and control of both FAdV-4 and FAdV-8a.

**IMPORTANCE:**

Epidemiological data reveal that FAdV-4 and FAdV-8a are the dominant serotypes of FAdVs in the poultry industry in China. Although three commercial inactivated vaccines against FAdV-4 have been licensed in China, the bivalent vaccine against both FAdV-4 and FAdV-8a is not available. Here, we used CRISPR-Cas9 and Cre-LoxP system to generate a recombinant virus FAdV4-F/8a-rF2 expressing the Fiber of FAdV-8a. Notably, FAdV4-F/8a-rF2 was highly attenuated and could provide efficient protection against both FAdV-4 and FAdV-8a in the chicken infection model, highlighting the applaudable application of FAdV4-F/8a-rF2 as a novel live-attenuated bivalent vaccine against the diseases caused by the infection of FAdV-4 and FAdV-8a.

## INTRODUCTION

Fowl adenoviruses (FAdVs) are non-enveloped, double-stranded DNA viruses belonging to the family *Adenoviridae*, genus *Aviadenovirus* (1). Currently, FAdVs are classified into five species (A–E) and 12 serotypes (1–7, 8a, 8b, and 9–11) based on genomic criteria and serum cross-neutralization tests (2, 3). FAdVs have spread globally with a wide infection spectrum, including chickens (4), ducks (5), geese (6), ostriches (7), and pigeons (8, 9). Among them, chickens are the most vulnerable. Chickens infected with particular FAdVs generally show different clinical syndromes, among which, FAdV-2/11 of species D and FAdV-8a and FAdV-8b of species E are responsible for the inclusion body hepatitis (IBH), while FAdV-4 of species C is responsible for the hepatitis-hydropericardium syndrome (HHS).

Since the outbreak of HHS caused by FAdV-4 prevailed in China in 2015, the FAdV-4 was the dominant serotype in domestic poultry flocks. Between 2015 and 2016, Niu et al. isolated 105 FAdV strains collected from IBH and HHS cases in China and found that 64.8% of the isolated strains belonged to FAdV-4, while only 0.95% of these isolates were identified as FAdV-8 (10). Notably, Chen et al. isolated 155 FAdV strains from diseased chickens between 2015 and 2018 and found that 79.4% (123 out of 155) and 13.5% (21 out of 155) of these isolates were identified as FAdV-4 and FAdV-8a, respectively (11), indicating that there is an increasing trend of FAdV-8a infection. The infection of FAdV-8a alone cannot cause severe disease (12 – 14); however, the outcome may become complicated when co-infected with other pathogens or other serotypes of fowl adenoviruses (14, 15), which raises concerns for the prevention of FAdV-8a.

To date, many efforts of vaccine development against FAdVs focus on the subunit recombinant proteins, and the Fiber protein has been repeatedly validated as an ideal antigen with high protective efficacy against different FAdV-induced diseases in chickens (16 – 19). Previously, De Luca et al. (19) and Schachner et al. (20) have demonstrated that either wild-type Fiber or chimeric Fiber derived from FAdV-8a could provide protection against homologous challenge; however, live attenuated vaccines against FAdV-8a have not been reported. Given that FAdV-4 is still an important and prevailing strain in domestic poultry flocks, we generated the recombinant virus FAdV4-F/8a-rF2 with Fiber-2 of FAdV-4 substituted with Fiber of FAdV-8a through the platform constructed previously (21). The animal experiment showed that the recombinant virus was not only highly attenuated but also could provide efficient protection against both FAdV-4 and FAdV-8a.

## RESULTS

### Construction and characterization of the recombinant virus FAdV4-F/8a-rF2

**Fig 1:**
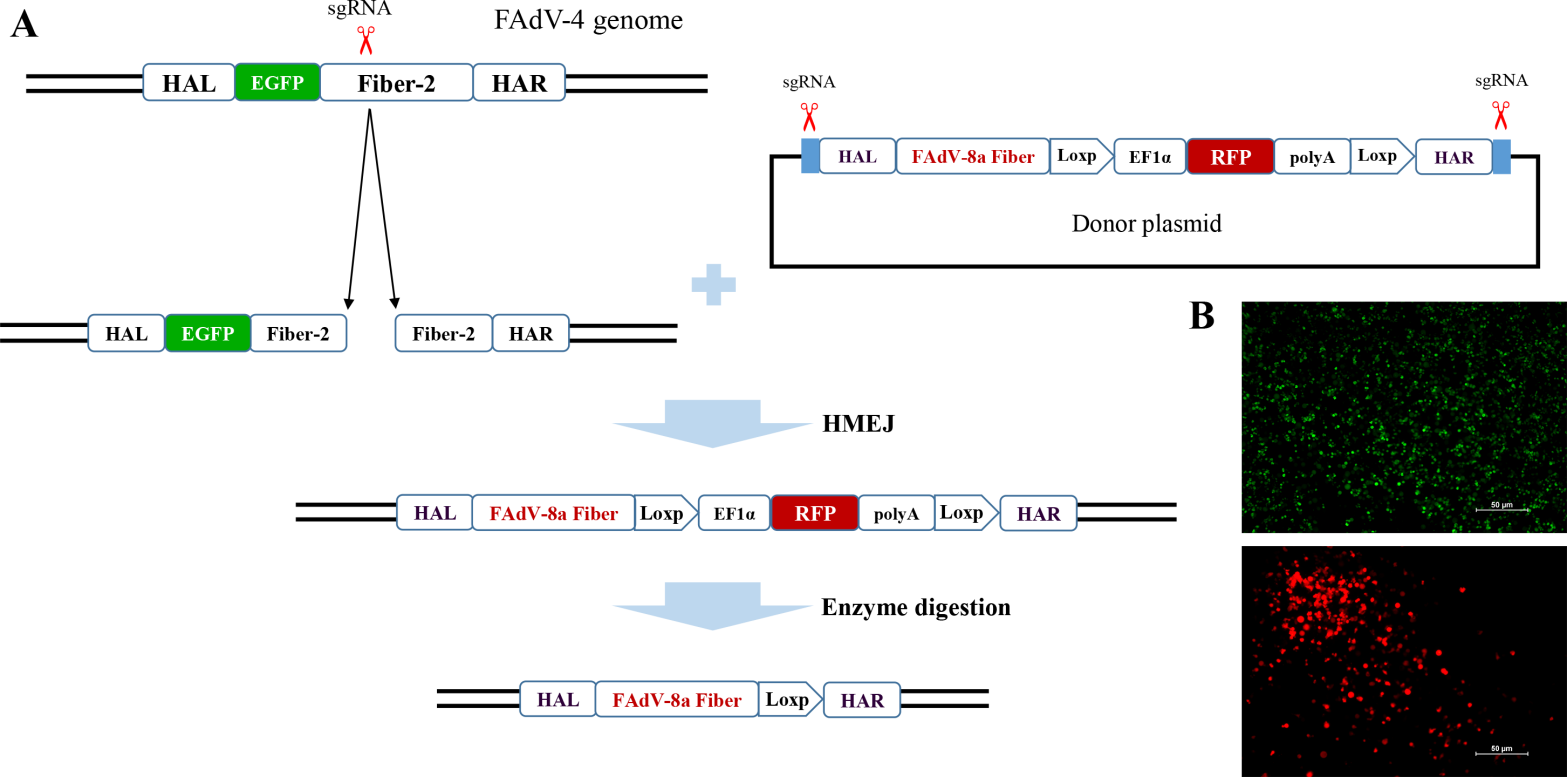
Rescuing of the recombinant virus FAdV4-F/8a-rF2. (**A**) Schematic presentation for the construction of the recombinant virus. (**B**) After the cell supernatant was inoculated into LMH cells, the EGFP indicating the parental virus FA4-EGFP and the RFP indicating the recombinant virus FAdV4-F/8a-rF2 could be viewed in the same vision field before purified.

**Fig 2:**
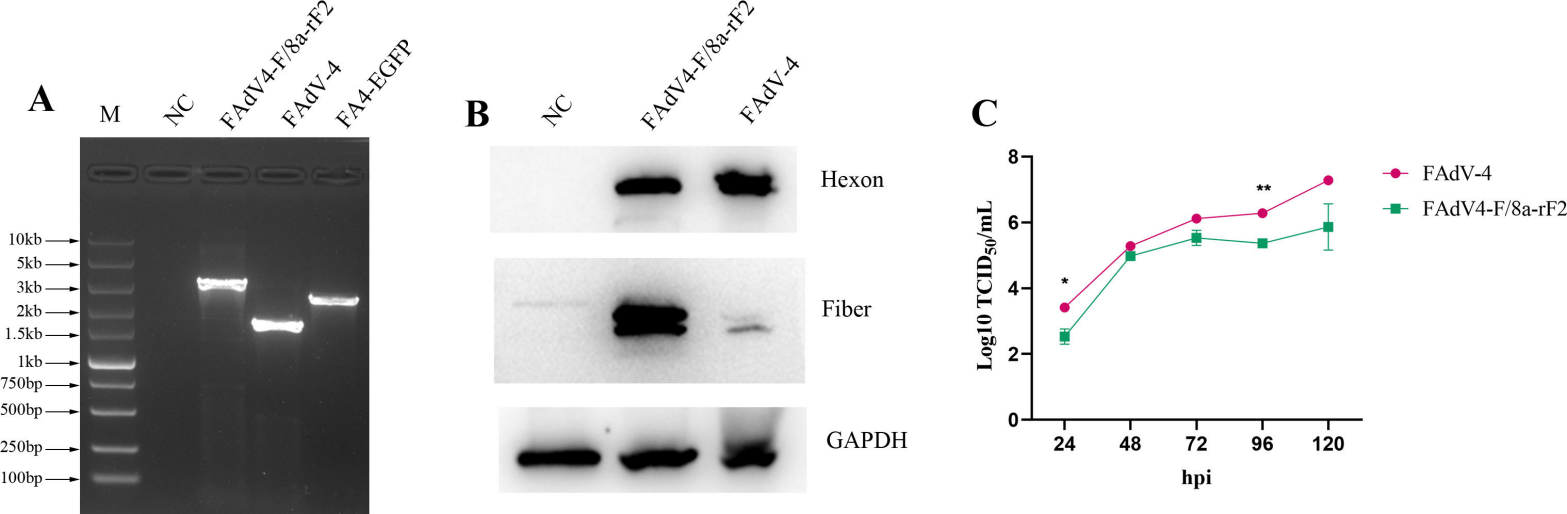
Identification of the recombinant virus FAdV4-F/8a-rF2. (**A**) PCR identification of the recombinant virus FAdV4-F/8a-rF2. After several rounds of purification, the viral DNA of the recombinant virus containing the RFP expression cassette was extracted and identified using the primers listed in Table 1; the wild-type FAdV-4 and parental virus FA4-EGFP were used as control. (**B**) Western blot analysis of the recombinant virus FAdV4-F/8a-rF2. LMH cells were infected with the recombinant virus FAdV4-F/8a-rF2; the expression of FAdV-8a Fiber in the LMH cells was examined by Western blot using the pAb against FAdV-8a. (**C**) Growth curve of the recombinant virus FAdV4-F/8a-rF2. LMH cells were infected with the recombinant virus FAdV4-F/8a-rF2 and FAdV-4, the supernatants were collected at indicated time points and titrated by TCID_50_, and the image was graphed using the GraphPad Prism 8 software.

To construct the recombinant virus expressing the Fiber protein of FAdV-8a using the FAdV-4 vector, the Fiber-2 of FAdV-4 was replaced with the Fiber of FAdV-8a through the double-fluorescence platform as shown in Fig. 1A. Briefly, sgRNAs and donor plasmid were co-transfected into LMH cells and subsequently infected with the FA4-EGFP, which expresses the EGFP-Fiber-2 fusion proteins. The recombinant virus expressing the RFP was observed through fluorescence microscopy. As shown in Fig. 1B, the RFP plaques indicating the recombinant virus and the EGFP indicating the parental virus could be observed in the same vision field, demonstrating that the recombinant virus was successfully rescued and named FAdV4-F/8a-rF2. The recombinant virus was then purified through the pickup of the RFP plaque and limited dilution assay. As shown in Fig. 2A, the PCR-specific band with the expected molecular weight (lane 3) demonstrated that the recombinant virus containing the RFP expression cassette was successfully purified, and the sequencing results further confirmed the substitution of FAdV-4 Fiber-2 with FAdV-8a Fiber as expected. The RFP expression cassette was then removed immediately after purification and sequencing. The sequencing results showed that the Fiber of FAdV-8a was inserted into the genome of FAdV-4 exactly as expected without any additional mutation, deletion, or insertion.

To identify the expression of the FAdV-8a Fiber, LMH cells were infected with the recombinant virus FAdV4-F/8a-rF2 and wild-type FAdV-4 and detected by Western blot with polyclonal antibody (pAb) against FAdV-8a. As shown in Fig. 2B, the abundant Fiber protein of FAdV-8a was easily detected in the cells infected with the recombinant virus FAdV4-F/8a-rF2, indicating that the Fiber of FAdV-8a was well-expressed by the FAdV4-F/8a-rF2. To further evaluate the replication capacity of the recombinant virus FAdV4-F/8a-rF2, LMH cells were infected with the recombinant virus and wild-type FAdV-4, respectively, the supernatants were then collected and titrated by TCID_50_. As shown in Fig. 2C, the recombinant virus FAdV4-F/8a-rF2 replicated in the same manner, but at a slower rate compared to the wild-type FAdV-4. All the results showed that the recombinant virus FAdV4-F/8a-rF2, in which the Fiber-2 of FAdV-4 was replaced by Fiber of FAdV-8a, was successfully rescued, and the Fiber of FAdV-8a was efficiently expressed.

### FAdV4-F/8a-rF2 was highly attenuated to SPF chickens

**Fig 3:**
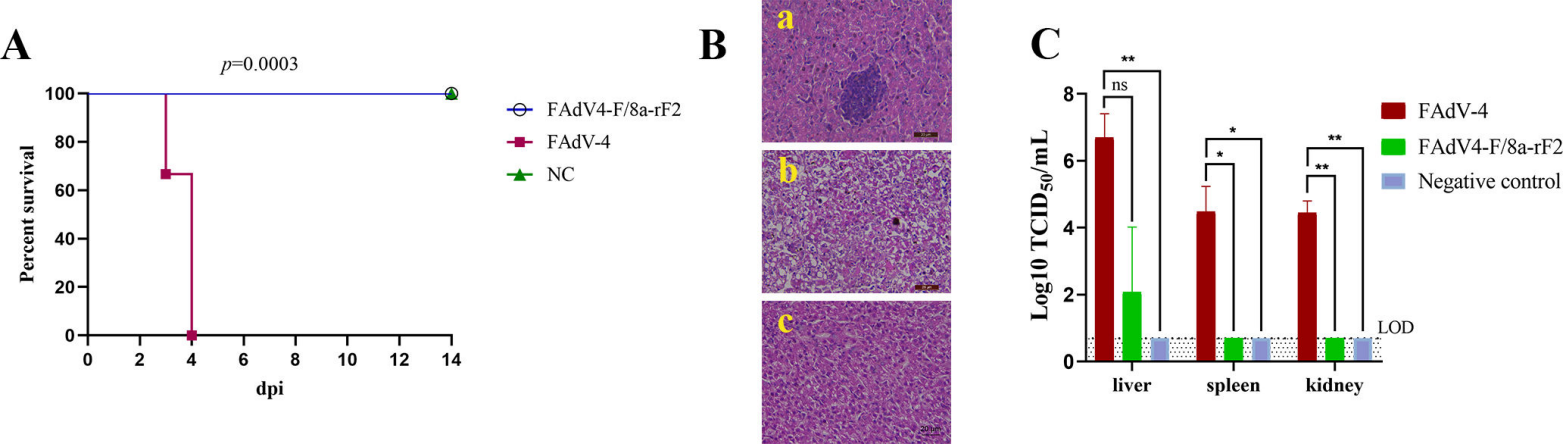
The recombinant virus FAdV4-F/8a-rF2 was highly attenuated. SPF chickens were infected with the recombinant virus FAdV4-F/8a-rF2 (*n* = 16) or the wild-type FAdV-4 (*n* = 6) at the same dose; the chickens inoculated with a cell culture medium containing 1% FBS were set as negative control (*n* = 16), and all the chickens were monitored for 14 days. (**A**) The survival curve of the infected chickens was graphed using the GraphPad Prism 8 software; the *P* value was calculated by Log-rank test. (**B**) At 4 dpi, chickens (*n* = 2) were sacrificed and the liver tissues were collected for further histopathological analysis. a—the liver of chicken from the vaccinated group. b—the liver of chicken from the FAdV-4 group. c—the liver of chicken from the negative control group. (**C**) The viral titers of the liver, spleen, and kidney collected and titrated by TCID_50_ and graphed using the GraphPad Prism 8 software.

To assess the pathogenicity of the recombinant virus FAdV4-F/8a-rF2, SPF chickens were inoculated with the recombinant virus FAdV4-F/8a-rF2 and wild-type FAdV-4 at the same dosage and monitored for 21 days. As shown in Fig. 3A, the chickens inoculated with FAdV-4 all died within 4 days, while those inoculated with the recombinant virus FAdV4-F/8a-rF2 all survived without any clinical symptoms during the period under observation. After necropsy, classical HHS was observed in the chickens inoculated with wild-type FAdV-4, while no gross lesions were observed in those inoculated with the recombinant virus FAdV4-F/8a-rF2. The histopathological analysis further confirmed that the chickens in the FAdV-4 group presented severe inclusion body hepatitis, degeneration, and necrosis in liver tissues, whereas the tissues in the vaccinated group were similar to the negative control group (Fig. 3B), only slight inflammation was observed in the liver of the vaccinated group. No pathological changes were observed in the spleen and kidney tissues of the vaccinated group (data not shown). To determine the viral loads in tissues, the liver, spleen, and kidney were collected from each group and titrated. As described in Fig. 3C, the viral loads of the liver from the FAdV-4 group could reach 10^6^ TCID_50_/mL, followed by spleen and kidney at the same level, while the viral loads of tissues from the vaccinated group were only detected in liver tissues, no virus was detected in the spleen and kidney. Collectively, our results demonstrated that the recombinant virus FAdV4-F/8a-rF2 was highly attenuated to SPF chickens.

### FAdV4-F/8a-rF2 efficiently protected against both FAdV-4 and FAdV-8a

**Fig 4:**
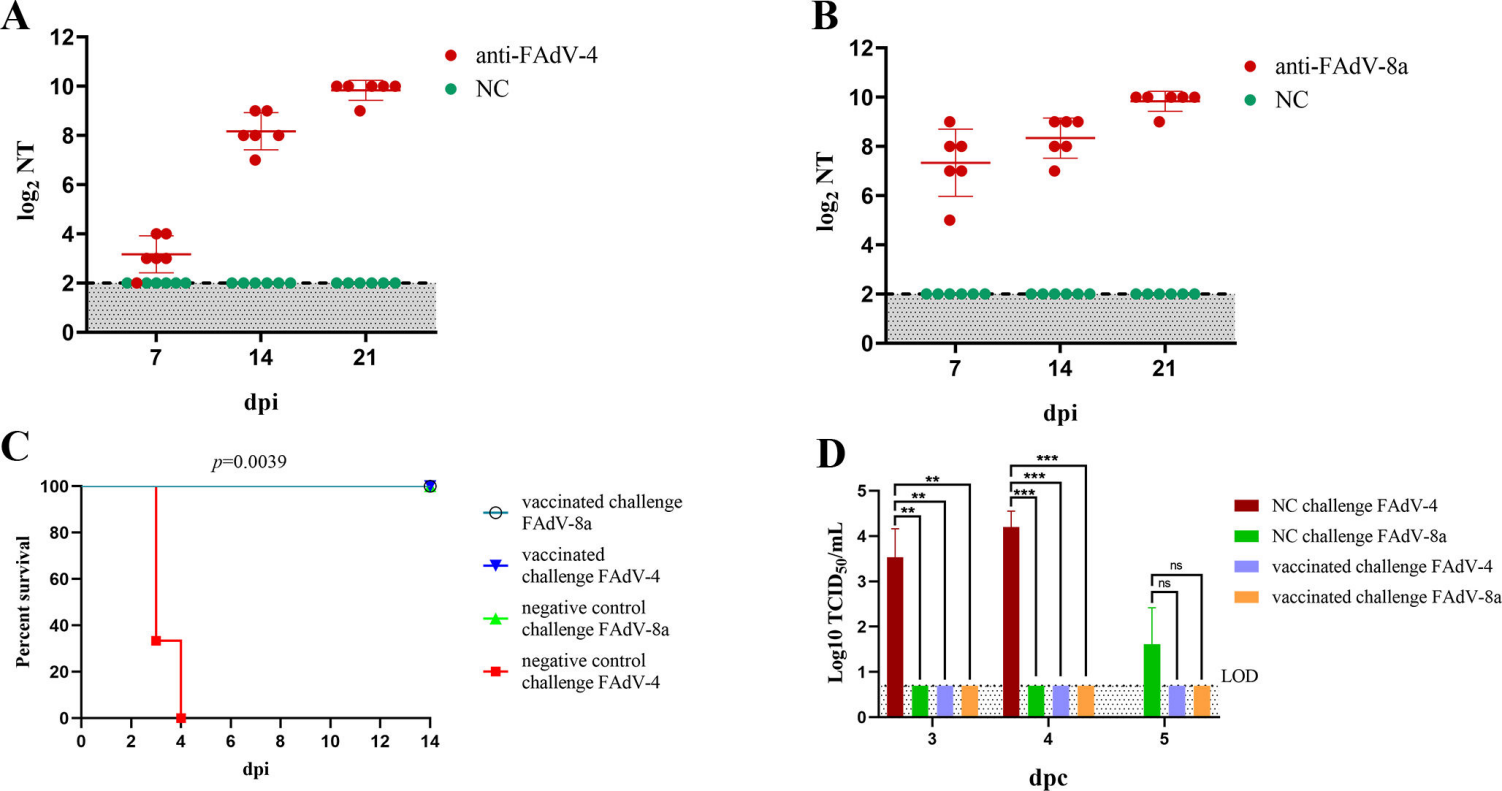
The recombinant virus FAdV4-F/8a-rF2 provided efficient protection. At 7, 14, and 21 dpi, the sera from chickens of different groups were collected for serological analysis. At 21 dpi, the survival chickens of the vaccinated group and negative control group were divided into four groups (*n* = 7 per group) and challenged with high doses of FAdV-4 and FAdV-8a. Morbidity and mortality were recorded daily. The cloacal swabs of different groups were collected at indicated time points and titrated through TCID_50_. (**A and B**) Neutralizing antibody titers against FAdV-4 and FAdV-8a. (**C**) Survival curve of the challenged chickens. (**D**) Viral shedding of chickens from different groups after challenge.

To evaluate the ability of the recombinant virus FAdV4-F/8a-rF2 on the induction of neutralizing antibodies against both FAdV-4 and FAdV-8a, the serum samples were collected from the vaccinated group and the negative group at indicated time points, and the neutralizing antibodies against FAdV-4 and FAdV-8a were determined. As described in Fig. 4A and B, the recombinant virus FAdV4-F/8a-rF2 induced high level of neutralizing antibodies against both FAdV-4 and FAdV-8a. The average neutralization titers against FAdV-4 were 9.3, 320, and 938.7 at 7, 14, and 21 dpi, respectively, and those against FAdV-8a were 218.7, 362.7, and 938.7 at 7, 14, and 21 dpi, respectively. However, the neutralizing antibodies of sera from the negative control group were all lower than 8.

To further evaluate the protective efficacy of FAdV4-F/8a-rF2, the chickens in the vaccinated group and negative control group were subsequently challenged with FAdV-8a or FAdV-4 at 21 dpi, respectively. As shown in Fig. 4C, the chickens in the negative control group challenged with FAdV-4 all died within 4 days, while the chickens in the vaccinated group challenged with FAdV-4 or FAdV-8a, and the negative control group challenged with FAdV-8a all survived. The viral shedding of chickens from different groups was then titrated, as shown in Fig. 4D. High viral titers were detected in the negative control group challenged with FAdV-4, while the titers of the negative control group challenged with FAdV-8a were relatively much lower, until 5 day post challenge (dpc) was the virus detected. Notably, no virus was detected in the vaccinated group challenged with FAdV-4 or FAdV-8a, as the high-level titers of neutralizing antibodies against both FAdV-4 and FAdV-8a. All these demonstrated that the recombinant virus FAdV4-F/8a-rF2 could efficiently induce immune response and provide protection against both FAdV-4 and FAdV-8a.

## MATERIALS AND METHODS

### Cells, viruses, and antibodies

The LMH cells (ATCC, CRL-2117) were maintained in DMEM/F12 medium (Gibco, NY, USA) supplemented with 10% fetal bovine serum (FBS, Lonsera, Shanghai, China) in a 5% CO_2_ incubator at 37°C. The recombinant virus FA4-EGFP expressing the EGFP-Fiber-2 fusion protein was generated and stored in our laboratory (22). The FAdV-8a strain AH720 was kindly provided by Professor Hongjun Chen (23). The wild-type FAdV-4 strain SD2015 and FAdV-8b strain JSSQ were isolated and stored in our laboratory (24). All the FAdVs were propagated in LMH cells maintained in DMEM/F12 medium containing 1% FBS. Monoclonal antibodies (mAb) 1B5 against hexon of FAdV were kindly provided by Professor Hongjun Chen (25), and pAbs against Fiber of FAdV-8a were collected from the mice immunized with purified GST-Fiber (FAdV-8a) and stored in our laboratory.

### Construction of donor plasmid and sgRNA

The donor plasmid with FAdV-8a Fiber was constructed based on our previous studies (26). Briefly, the tail of Fiber-2 of FAdV-4 and HA gene of H9N2 in previously constructed donor plasmid were deleted and subsequently replaced with full-length Fiber of FAdV-8a through the one-step clone. The primers used for the construction of donor plasmid are listed in Table 1. The sgRNAs targeting the Fiber-2 of FAdV-4 were constructed previously and stored in our laboratory.

**TABLE 1:** Primers used for the construction of donor plasmid for the identification of recombinant virus

PCR product	Sequence of primers (5′−3′)
Fiber of FAdV-8a	F: GGCCCCTAAAGCGACCTCGACTCCTCACGCCTTC
	R: CTTTCTAGACTCGAGTTATGACACGTCCGCAGTACTTAG
Linearized donor plasmid	F: TAACTCGAGTCTAGAAAGCTTGGATC
	R: GAGGTCGCTTTAGGGGCCCGGAGCATTGTTC
31616–33285	F: CTCCAACTGGTTTGACCAGAACG
	R: GTTGTATGATTGGACGCGGGAAC

### Rescue of the recombinant virus

The recombinant virus expressing the Fiber of FAdV-8a was rescued on LMH cells. First, 2 µg of the donor plasmid and sgRNA in each were co-transfected into LMH cells. At 10 hours post-transfection (hpt), the LMH cells were infected with FA4-EGFP at a multiplicity of infection (MOI) of 0.1 for 2 hours. Later, the supernatants were removed, and the LMH cells were maintained in a medium containing 1% FBS. At 48–72 hours post-infection (hpi), the supernatants of the LMH cells were harvested and centrifuged at 12,000 rpm, and the supernatants were then inoculated into the newly prepared LMH cells. Fluorescence was observed daily to confirm the existence of a recombinant virus expressing the RFP. The purification of the recombinant virus and removal of the RFP expression cassette were conducted as previously described (26). The DNA of the purified recombinant virus was then extracted to identify the purity through PCR. The purified recombinant virus was then verified by sequencing, Western blot, and indirect immunofluorescence assay (IFA).

### Growth curve of the recombinant virus

The LMH cells seeded in a 6-well plate were infected with the rescued recombinant virus or wild-type FAdV-4 at an MOI of 0.1 in duplicate, the supernatants were then collected at 24, 48, 72, 96, and 120 hpi, and stored at −80°C before use. The 50% tissue culture infectious dose (TCID_50_) was determined by serial dilution from 10^−1^ to 10^−8^ with four replicates of each concentration and subsequently inoculated into LMH cells seeded in the 96-well plate. At 96 hpi, the LMH cells were fixed and examined by IFA using the mAb 1B5 against the hexon of FAdV-4. The fluorescence plaques were counted, and the viral titers were then calculated by Reed-Muench method.

### PCR analysis of the recombinant virus

Viral DNA from the cell supernatant of infected LMH cells was extracted using TIANamp Genomic DNA Kit (Tiangen, Beijing, China), The primers were designed from the left and right homology arm, which can be used to identify the wild-type FAdV-4, the parental virus FA4-EGFP, and the recombinant virus, with the expected size of products approximately 1,600, 2,300, and 3,000 bp, respectively. The primers used for PCR identification are listed in Table 1.

### Immunofluorescence assay

LMH cells were fixed with pre-chilled acetone: ethanol (3:2 vol/vol) mixture for 5 min at room temperature (RT). After the mixture was removed and evaporated, the LMH cells were washed once with PBS and incubated with the mAb 1B5 against hexon of FAdV-4 for 45 min at 37°C in a thermostat water bath. After three washes with PBS, the LMH cells were incubated with secondary antibodies (goat against mouse IgG-FITC) for another 45 min at 37°C. After another three washes with PBS, the LMH cells were examined by fluorescence microscopy.

### Western blot

LMH cells were first infected with wild-type FAdV-4 or the rescued recombinant virus, and the LMH cells were collected at 48 hpi and lysed in lysis buffer with phenylmethanesulfonyl fluoride (PMSF), protease, and phosphatase inhibitors. The lysates were then boiled with protein loading buffer, separated on an SDS-PAGE gel, and transferred onto a nitrocellulose membrane. After blocking with blocking buffer, the membrane was incubated with indicated primary antibodies. After three washes with phosphate buffered saline with Tween-20 (PBST), the membrane was then incubated with correspondence secondary antibodies (goat against mouse IgG-HRP). After another three washes with PBST, the membrane was developed with chemiluminescent reagents and imaged with an automatic imaging system (Tanon 5200).

### Animal study

A total of 38 Babcock SPF chickens aged 2 weeks were divided into three groups designated as vaccinated group, negative control group, and FAdV-4 group. Chickens in the vaccinated group (*n* = 16) and the FAdV-4 group (*n* = 6) were inoculated with 1 × 10^6^ TCID_50_ of the recombinant virus FAdV4-F/8a-rF2 and wild-type FAdV-4 intramuscularly, respectively. The chickens in negative control (*n* = 16) were inoculated with a cell culture medium containing 1% FBS; all the chickens in different groups were inoculated with the same volume. After inoculation, the chickens were monitored daily, and the cloacal swabs were collected at indicated time points for viral titration. Two chickens in each group were sacrificed, and the liver, kidney, and spleen were collected for viral titration and histopathological examination. The serum was collected at 7, 14, and 21 dpi for serological analysis. At 21 dpi, chickens either in the vaccinated group or negative control group were randomly divided into two groups and designated as vaccinated challenge FAdV-8a group, vaccinated challenge FAdV-4 group, negative control challenge FAdV-8a group, and negative control challenge FAdV-4 group. Chickens in the four groups were challenged with 1 × 10^6^ TCID_50_ of FAdV-8a or wild-type FAdV-4 intramuscularly. After the challenge, the chickens were monitored daily, and the cloacal swabs were collected at indicated time points for viral titration.

### Histopathological examination

The liver, spleen, and kidney tissues were collected from dead or euthanized chickens and fixed with 10% formalin. The fixed tissues were embedded in paraffin and sectioned for examination after hematoxylin-eosin staining.

### Neutralizing antibodies determination

The serum was first inactivated at 56°C for 30 min and twofold serially diluted (start dilution 1:4). The diluted serum was then incubated with 100 TCID_50_/100 µL of indicated virus at 37°C for 1 hour and subsequently inoculated into LMH cells in 96-wells plate. After incubation for 2 hours, the mixture was removed, and the LMH cells were washed once and maintained in a maintaining buffer containing 1% FBS. After being cultured for 96 hours, the LMH cells were fixed and examined with IFA using the mAb 1B5 against the hexon of FAdV-4.

### Viral titration in organs and cloacal swabs

The tissues collected from sacrificed chickens were homogenized with PBS and centrifuged at 12,000 rpm for 10 min. The supernatants were then collected and treated with 10× penicillin and streptomycin for 1 h and stored at −80°C before use. The cloacal swabs collected at indicated time points were suspended with PBS and freeze-thawed three times. After centrifugation at 12,000 rpm for 10 min, the supernatants were collected and treated with 10× penicillin and streptomycin for 1 h. The virus-containing supernatants of organs and cloacal swabs were then serially diluted and inoculated into LMH cells. At 96 dpi, the infected LMH cells were fixed and examined by IFA using the mAb 1B5 against the hexon of FAdV-4 or pAb against Fiber of FAdV-8a. The viral titers were then calculated by the Reed-Muench method.

### Statistical analysis

All the results are presented as means ± standard deviation. The statistical analysis in this study was performed with a *t*-test or Log-rank test using GraphPad Prism 8 software. *P* value < 0.05 was considered significant. *, **, and *** indicate *P* values less than 0.05, 0.01, and 0.001, respectively.

## DISCUSSION

Fiber, penton, and hexon proteins are the major structural proteins on the virion of FAdV. Since FAdV-4 infection became epidemic in China, various vaccines based on the structural proteins have been developed to control and prevent the disease (17, 18, 27, 28). However, enforced vaccination against one serotype may cause a shift toward outbreaks of other serotypes. Epidemiological investigations show that the infection of other serotypes of fowl adenovirus, especially FAdV-8a, has significantly increased recently and chickens co-infected with some other pathogens have become common, which calls for the urgent development of multivalent vaccines against FAdV and other avian pathogens. So far, only subunit chimeric Fiber proteins (crecFib-4/11 and crecFib-8a/8b) and inactivated chimeric FAdV-4 with FAdV-8b Fiber were developed (20, 29 – 31); however, the chimeric FAdV-4 with Fiber of FAdV-8a has not been developed yet. Our previous studies have demonstrated that Fiber-2 of FAdV-4 can serve as an efficient foreign gene insertion site for the generation of attenuated recombinant FAdV-4 (32). In this study, we have successfully generated the recombinant virus FAdV4-F/8a-rF2, which replaces the Fiber-2 of FAdV-4 with the Fiber of FAdV-8a, using the recombinant virus platform constructed previously.

Fiber-2 and hexon are reported to be closely related to the virulence of highly pathogenic FAdV-4 (33), while Fiber-1 is responsible for the binding with cellular receptor and mediates viral infection (34, 35). Recently, several recombinant viruses were constructed by manipulating Fiber-1 or Fiber-2 of FAdV-4. The recombinant virus either inserted the Fiber of FAdV-8b between Fiber-1 and Fiber-2 of FAdV-4 (31) or replaced Fiber-1 of FAdV-4 with the Fiber of FAdV-8b (30), and showed pathogenicity to SPF chickens. However, the recombinant FAdV-4 with the full-length Fiber-2 replaced with EGFP was highly attenuated in SPF chickens, and the recombinant virus showed low replication capacity *in vivo* and *in vitro* (32). As Fiber-1 is involved in the tissue tropism of FAdV-4 (36), the FAdV-4 without Fiber-2 may lose virulence but not tropism. All these findings highlight the role that Fiber-2 plays in the virulence and viral replication of FAdV-4. In this study, the Fiber-2 of FAdV-4 was replaced with the Fiber of low pathogenic FAdV-8a, while the Fiber-1 of FAdV-4 remained intact. Therefore, the recombinant virus could infect LMH cells and efficiently replicate *in vitro*; however, the Fiber from low pathogenic FAdV-8a failed to support the efficient replication of the recombinant virus *in vivo*, and no clinical symptoms or inclusion bodies were observed in the infected chickens inoculated with a high dose of the recombinant virus FAdV4-F/8a-rF2. On the other hand, adenoviruses were highly effective in the expression of foreign genes (37), and live attenuated vaccines could efficiently induce cellular and humoral immune responses (38). Therefore, although inactivated rFAdV-4-fiber/8b and FA4-F8b could provide efficient protection against both FAdV-4 and FAdV-8b, the live attenuated could be an alternative choice for better control and prevention of FAdVs. However, the risk of the live attenuated vaccine should not be neglected, as the recombinant virus may adapt and replicate faster after continuous passage, and there is a possibility of turning back to virulence. Besides, situations may become complex when chickens are infected with immunosuppressive pathogens; therefore, the live attenuated vaccine should be thoroughly evaluated before application.

Fiber was the first component of adenovirus to interact with host cells, and FAdV-4 infection is initiated by the interaction of Fiber-1 with Coxsackievirus and Adenovirus Receptor (35). Previous studies have shown that the shaft and knob domain of Fiber-1 not only block the infection of FAdV-4 (34) but also confer the superinfection resistance against FAdV-8b (39), indicating that FAdV-4 and FAdV-8b may share the same cellular receptor. In this situation, the clinical polyclonal antibodies against FAdV-4 may show the ability to neutralize FAdV-8b. However, the neutralizing antibody test revealed that the sera collected from the vaccinated group failed to neutralize FAdV-8b (data not shown), indicating that the mechanism of the shaft and knob domain of Fiber-1 of FAdV-4 on blocking FAdV-8b infection needs further investigation. Notably, our previous study also demonstrates that the FA4-F8b expressing the Fiber of FAdV-8b can efficiently protect against both FAdV-4 and FAdV-8b but not FAdV-8a (31). To construct a recombinant virus that provides simultaneous protection against FAdV-4, FAdV-8a, and FAdV-8b, a chimeric Fiber with two serotypes’ neutralizing epitopes may be required, as Schachner et al. have previously demonstrated (20).

In summary, a novel recombinant virus FAdV4-F/8a-rF2 expressing the Fiber of FAdV-8a was constructed through the replacement of Fiber-2 of FAdV-4 with the Fiber of FAdV-8a by using the template virus FA4-EGFP. The recombinant virus FAdV4-F/8a-rF2 replicated slightly slower than the wild-type FAdV-4 *in vitro* but was non-pathogenic to SPF chickens. Moreover, the recombinant virus could induce a high level of neutralizing antibodies against both FAdV-4 and FAdV-8a and provide efficient protection against both FAdV-4 and FAdV-8a, highlighting that the recombinant virus FAdV4-F/8a-rF2 generated here can be used as an attenuated vaccine candidate for the prevention and control of diseases caused by the infection of FAdV-4 and FAdV-8a.

## Data Availability

The datasets used and analyzed in the study are available from the corresponding author upon reasonable request.
